# Legal or Illegal, Cannabis Is Still Addictive

**DOI:** 10.1089/can.2015.29004.rtd

**Published:** 2016-01-01

**Authors:** Daniele Piomelli, Margaret Haney, Alan J. Budney, Pier Vincenzo Piazza

**Affiliations:** ^1^ Department of Anatomy and Neurobiology, School of Medicine, University of California–Irvine, Irvine, California.; ^2^ New York State Psychiatric Institute–Columbia University Medical Center, New York, New York.; ^3^ Addiction and Health Research, Center for Technology and Behavioral Health, Geisel School of Medicine, Dartmouth College, Lebanon, New Hampshire.; ^4^ Magendie Neurocenter, INSERM U862, Bordeaux, France.

**Clinical and experimental work spanning the last two decades has demonstrated that Cannabis-derived drugs such as Cannabis can cause addiction. Yet, this evidence has yet to permeate the scientific community and the public. We have asked three leaders in the field to discuss this.**

**Dr. Daniele Piomelli:** **Let's start from the beginning. What are the key features that define drug addiction? Dr. Piazza, would you mind taking the lead on this one?**

**Dr. Pier Vincenzo Piazza:** Addiction is a vague concept that is generally used to define a pathological drug taking. There are different levels of severity of this pathological behavior. The mild condition is mostly characterized by an escalation in drug intake that goes from a sporadic recreational drug use to a more sustained and regular drug intake. This behavior starts becoming a problem for the subject who can engage in risk-taking behaviors, shows drug-related health problems, and has difficulties discontinuing drug use. However, at this stage, the behavior of the subject still remains mostly organized. The subject can almost keep a normal life.

The later phase, what normally people refer to as addiction, is characterized by loss of control on drug use. Loss of control on drug taking can be defined by three key features. The subjects cannot control the amount of drug that is taken. The subjects spend more and more time looking for the drug or engaged in activity that allows acquiring and using drugs. The subject cannot stop using drugs despite the consciousness of adverse consequences. In other words, drug seeking becomes the only behavior that really controls the life of the individual and a “normal” life becomes impossible.

**Dr. Daniele Piomelli:** **Thank you. That was very clear. Does anybody want to add anything to Dr. Piazza's remarks?**

**Dr. Alan J. Budney:** I would just reiterate for the audience that the last phase of addiction is the extreme end of the disorder. Addiction, as was just described, ranges from mild to severe. There are many people with serious problems that fall in the middle of that range. It is very important to recognize this range of problems or phases when educating the public about addiction, particularly when substances like Cannabis are the topic.

**Dr. Margaret Haney:** I agree, and I might be jumping ahead but part of society's slow recognition of Cannabis use disorder (CUD), and the possibility of addiction, is the severity of the addiction itself. We will talk about evidence for there being a CUD, but in studying cocaine and alcohol and other drugs of abuse, in my opinion, CUD does not result in the same extreme levels of behavior that one sees with addiction to other drugs of abuse. And we do not want to lose this message when communicating the risks of Cannabis. We have to communicate the reality and what the real consequences of daily Cannabis use are and not exaggerate them.

**Dr. Daniele Piomelli:** **In your answer, Dr. Haney, you used the term “Cannabis use disorder (CUD).” In fact, when discussing addiction, the 5th edition of *Diagnostic and Statistical Manual of Mental Disorders* (*DSM*), edited by the American Psychiatric Association, prefers to use the term “substance-related disorder” over “addiction.” Could you perhaps explain to the nonspecialist reader, Dr. Haney, the meaning of a substance-related disorder?**

**Dr. Margaret Haney:** I think Dr. Budney might have better insight on this because he served on the *DSM* committee. However, the term “addiction,” I believe, was considered to be pejorative and so the *DSM* came out with use disorders as another way to describe the behavior without addict or addiction. Dr. Budney, is that your understanding as well?

**Dr. Alan J. Budney:** Yes, I guess that was a part of the decision process. The fact is that the term “addiction” was not part of *DSM* 3rd or *DSM* 4th edition either. The terms “substance abuse” and “substance dependence” were used to designate substance addiction disorders. The term “addiction” has not been included as a categorical label. And I think to some degree that relates to what Dr. Haney just described; some people consider it a pejorative term, but I think it is also because the term “addiction” is commonly used to refer to represent only the severe range of substance use disorders.

The change to “use disorder” reflects multiple considerations but is meant to indicate that a person can have a problem with substances that does not necessarily coincide with the extreme image that many lay people and scientists envision when they think “addiction,” which is wildly uncontrolled, compulsive behavior, similar to the last phase described earlier by Dr. Piazza.

A person can have problems with substances that are very substantial all along a continuum, and neither clinicians nor scientists have highly effective ways to distinguish among those severity levels, and designate a cutoff for addiction that is clearly discriminated from less severe forms of the disorder. The range of symptoms and consequences we observe in clinical settings is quite large. Nevertheless, we have not yet developed an effective marker to designate meaningful distinctions across individuals. The *DSM* 5th edition provides a method for designating severity level by summing the number of diagnostic criteria displayed; however, no matter what the severity level, the person still receives a diagnosis of a use disorder—mild, moderate, or severe.

**Dr. Pier Vincenzo Piazza:** I just wanted to add one thing. Probably one of the most interesting points of the *DSM* 5th edition has been to officially recognize what Dr. Budney was saying: there is a continuum in the severity of the disorders related to drug use, although scientists, clinicians, and the general public conceptualize addiction as the last more severe stage. This implies that also mild use disorders need a therapeutic intervention and can give serious problems to the individual without the need to reach the extreme state. So, I completely agree with what has been said before.

**Dr. Daniele Piomelli:** **What are the specific features of CUD?**

**Dr. Alan J. Budney:** The features of CUD are the same as the features of all substance use disorders according to the *DSM* 5th edition. There is a list of 11 criteria, and they encompass the range of signs and symptoms that can be experienced, including physiological signs like tolerance and withdrawal to Cannabis, continuing to use Cannabis despite the person knowing he or she has problems being caused by Cannabis, and recurrent use in situations that might be hazardous or dangerous like driving a car while high on Cannabis. Other signs include using to such excess that Cannabis seems to takes over one's life, and healthy behaviors like work and recreation and positive relationships are harmed, ignored, or greatly reduced. Experiencing strong and frequent cravings to use, and using more Cannabis than one plans to use or for a much longer time than was planned are also common features of the disorder. Last, people who develop problems with Cannabis may have repeated desire to cut down or quit, but end up going back to using the same amount or more. Some scientists have tried to identify, statistically, hallmark signs or symptoms of CUD that differentiate it from other substance use disorders, but they have not been successful. Essentially, CUD manifests in the same way as other substance use disorders but the difference may be in the magnitude of severity of each of the signs and symptoms that are experienced.

One example of this is the difference in severity of withdrawal symptoms experienced by those who abruptly stop using opiates versus Cannabis. Heavy Cannabis users who stop experience withdrawal symptoms that may be somewhat similar to tobacco withdrawal symptoms, but they typically do not approach the severity nor have the clinical implications of the withdrawal experience by many opiate users.

**Dr. Daniele Piomelli:** **It seems that most specialists in the field agree that Cannabis is addictive. If you had to choose one piece of evidence, either clinical evidence or animal experiment evidence, in support of this conclusion, which one would you pick?**

**Dr. Margaret Haney:** One of the key features for me is demonstrating that there is a pharmacologically specific withdrawal from Cannabis use. And this is the work that both Dr. Budney and I have done for many years. We can demonstrate that daily smokers go through a time-dependent and pharmacologically specific withdrawal when they abstain from Cannabis. If you replace Cannabis use with low amounts of tetrahydrocannabinol (THC), you can reverse this withdrawal phenomenon.

I think another really important feature is the clinical data showing how high relapse rates are with Cannabis. Although Cannabis may have lower abuse liability than other drugs like cocaine or nicotine, once somebody has developed a dependence on the drug, then quitting becomes extremely difficult. So again, the relapse rates for Cannabis are as high as other drugs of abuse. I think it is important for other scientists and for the public to be aware that it might not be as easy to develop dependence, but once you have it, then quitting will become extremely difficult.

**Dr. Alan J. Budney:** I would second that. If I had to pick out the “smoking gun” to convince the public and the scientific world that CUD is real, then it would be the data from clinical epidemiological research. Certainly, the evidence from behavioral pharmacology, clinical pharmacology, and the neuroscience research is important and robust. However, if you look at prevalence rates in the general population who report substantial problems with different types of substances and the rates of substance users that enroll in treatment, and relapse rates following quit attempts, the data on CUDs are remarkably similar to the other substance use disorders. So, as Dr. Haney just pointed out, I do not think there is any argument to counter the fact that, for a substantial number of people, Cannabis use causes similar and substantial problems that are comparable to other types of drugs that we all agree have addictive potential.

**Dr. Pier Vincenzo Piazza:** I would use the same smoking gun as Dr. Budney but with a small statistical precision that is provided by patient's demand. If you take the statistics from the four countries for which we have the best surveys, Australia, Canada, the United States, and the European Union, over the last two years Cannabis represents the highest new entries for treatment in specialized centers. The statistics are higher now for Cannabis than alcohol, which was number one before.

Since these four countries have very different rates of referral of patients by the judicial system, these figures really mean that patients experience a discomfort high enough to spontaneously seek treatment. The negative perception of the patient of her or his condition, reflected by the demand to be treated, I think, is a very important demonstration of the serious behavioral problems associated with CUD.

**Dr. Margaret Haney:** I definitely concur. And when I speak on Cannabis to the public or to the scientific community, I like to make that distinction because a subset of treatment seekers in the United States are mandated to treatment. Yet the important thing to highlight, as Dr. Piazza mentioned, is the number of Cannabis users seeking treatment on their own initiative. These are adults seeking treatment on their own initiative.

**Dr. Pier Vincenzo Piazza:** Absolutely. In France, for example, as well as in many other European countries, referral from the judicial system is very low. Nevertheless, the demand for treatment for CUD is now the highest of all drugs, legal and illegal.

**Dr. Daniele Piomelli:** **I would like to add that there is also ample evidence from animal work that rodents and primates can become dependent on the main component of Cannabis, THC. But moving forward, what is curious is that we now accept the concept that Cannabis is addictive, but for many years we have been told that it was not. Why is it that, for so long, the scientific community failed to recognize the addictive properties of Cannabis?**

**Dr. Margaret Haney:** I have been speaking about Cannabis addiction for 20 years and was met by full-on boredom for the first 15 years because I felt that scientists, like the public at large, just viewed Cannabis as a benign compound not too different from caffeine in a way. There was not any interest, but now that has changed.

I think one of the factors is that, because THC is lipophilic, and so long-lasting, withdrawal takes quite a while to manifest. In humans it usually takes about 24 hours, which is unlike cigarettes, where if an individual is dependent on nicotine, he or she cannot go a couple of hours without experiencing withdrawal. A heavy Cannabis user, by contrast, has to go quite a while before experiencing withdrawal, and so it was not quite as obvious to people that withdrawal existed.

**Dr. Alan J. Budney:** I would like to add a couple of other factors that may have contributed to this perception. For a long time, scientists had great difficulty showing in animal experiments that animals would self-administer Cannabis or THC-type compounds. Much of the hallmark work in the early days of addiction research was definitely from the animal labs. So, not being able to demonstrate Cannabis self-administration or withdrawal in animals made it difficult to claim it was an addictive substance like the opioids or cocaine. Scientists did finally solve these issues and now have clearly demonstrated Cannabis self-administration and withdrawal in the animal lab.

In addition to that, I think that our original topic today, which is the difficulty in defining and agreeing upon a definition of addiction, contributed and perhaps still plays a role in the perception that Cannabis is not addictive. Many scientists and much of the general population think of addiction in extremes, with severe opiate withdrawal and alcohol withdrawal being the hallmark indicator of true addiction. As mentioned earlier, scientists did not have clear evidence of Cannabis withdrawal that mirrored that of opiate or alcohol withdrawal. Moreover, many of those that have experience with using Cannabis, do not get addicted, develop problems, or experience withdrawal. Although the same is true for those who have used alcohol or even opiates, for reasons that are not completely clear, the personal experience of those who used Cannabis and did not develop problems or experience withdrawal, seems to lead to the perception that Cannabis is not a substance that others can become addicted to.

**Dr. Pier Vincenzo Piazza:** I think there could be also, paradoxically, a generational problem. Cannabis contains several active compounds but principally two: THC, which mediates most of Cannabis effects, and cannabidiol, which is an antagonist of THC. When you look at the Cannabis of the 1970s, which is basically what scientists were smoking when they were young, there was almost a 50/50 percentage between THC and cannabidiol. What we know now is that, since cannabidiol is an antagonist of THC, the greater the ratio between THC and cannabidiol the greater the risk for Cannabis to be addictive.

Recently, we have seen appearing strains of Cannabis in which THC concentrations are increasing. Now, we are up to a 5- to 10-fold difference in favor of THC, making Cannabis more addictive. This is also reflected in the statistics of addictive liability of Cannabis. The number of individuals moving from Cannabis use to CUD has gone from 8% to 15% in the last 15 years. So, one element that could have contributed to the increased recognition of the addictive potential of Cannabis is that the Cannabis available today, which contains higher concentrations of THC, has increased the potential to cause CUD.

**Dr. Margaret Haney:** I think it is important to know that there is probably a European and a North American difference because in the United States in the 1970s Cannabis was roughly 1.7% THC and had very low levels of cannabidiol. Now it is on average 12–20% THC but still very low levels of cannabidiol, whereas in Europe, hashish was primarily smoked and that, I understand, is about 50/50 cannabidiol and THC. So, we never had much cannabidiol here in the United States. There certainly is a cultural and a geographic difference in the way that Cannabis was smoked in the early days. Now we are pretty much caught up with each other, I think.

**Dr. Pier Vincenzo Piazza:** I agree with your comment, since in any case the final result is that we observe higher and higher concentrations of THC in Cannabis today and a final much higher THC/cannabidiol ratio.

**Dr. Margaret Haney:** Yes, definitely.

**Dr. Daniele Piomelli:** **This discussion is really important. It brings me to ask another related question that may be a little hard to answer right now. And it is the question at the heart of a lot of people who use Cannabis or wonder whether to use Cannabis or not. How addictive is Cannabis? Is it more addictive than, say, tobacco or alcohol? Is it less addictive? Is this question even correctly asked? Is there a better way to ask it?**

**Dr. Alan J. Budney:** I think that your series of questions describes the problem with the question. First, to answer the question, we have to agree on a working definition of addictive and addiction. And as we discussed earlier, it is not a very well-defined construct.

For example, are you referring to the severity of the problem that develops, or is it the probability of acquiring a problem? Is the comparison how likely or probable one is to develop a problem if he or she tries it? Or another comparison could be, how hard is it to quit if you develop a problem? All these things come into play. On top of that, one might ask, when comparing, should consideration be given to the environmental context? Many factors other than just the pharmacology of the substance and how it is administered contribute to how “addictive” or how likely it is that a person will develop a problem, which I think is what we are really interested in. And in my opinion those factors cannot be divorced from the questions about addictive potential. If alcohol, for example, cost $40 for a pint of beer, and high-potency Cannabis cost $30.00 an ounce, and both were legal and freely available, we would observe large differences in the use and the development of problems with each substance than if the costs were $2.00 a pint and $450 an ounce and one was legal and the other not. You see what I am getting at? This is a tricky and complex question. I will leave it to my colleagues to tell you the true answer to the question.

**Dr. Margaret Haney:** Well, the relative abuse liability of different drugs is partly assessed by looking at the epidemiological data: if you try a drug once, how likely are you to go on and develop a use disorder? But that, of course as Dr. Budney says, is confounded by societal factors. What we are seeing epidemiologically is changes in Cannabis use. A recent article in *JAMA Psychiatry* reported that in the United States attitudes toward Cannabis have shifted tremendously in the past few years. Cannabis use rates are way up, and therefore more people will develop a CUD.^1^

My opinion is that Cannabis has a lower abuse liability than something like cocaine. Nonetheless, because of the more permissive societal attitude toward Cannabis, a larger number of people are using this drug than before, and so more people will develop a problem with it. Thus, even if Cannabis has a lower abuse liability, the sheer number of people using it will result in a large number of people with a use disorder.

**Dr. Pier Vincenzo Piazza:** If we try to express abuse liability in numbers, the abuse liability for Cannabis, in the sense of the probability that you have to develop CUD if you smoke once, is between 10% and 15%, depending on the survey you look at. In comparison, cocaine, alcohol, and heroine are in a range that is between 20% and 25%. Nicotine has the highest abuse liability with a probability of 33% to induce dependence.

However, I believe that abuse liability should also be measured by a second factor that is how easy it is to quit if you have developed a substance use disorder. My understanding, and probably Dr. Alan Budney can say more, is that stopping Cannabis use, if you have developed CUD, is not easier than other drugs.

Another element to be taken into account is that prevalence of Cannabis use is very, very high. Consequently, although a lower percentage of individuals using Cannabis will develop a substance use disorder, compared with individuals using other drugs, CUD is going to be the major drug-related problem in the next decade.

**Dr. Alan J. Budney:** I agree with Dr. Piazza and would like to emphasize a point so that our audience does not think we are going way overboard and engaging in reefer madness related to the severity of Cannabis addiction. All factors held constant, the pharmacology of opiates would probably produce a more severe addiction that would be more entrenched and harder to quit related to the opiates impact on the brain systems, euphoric experience, and the development of tolerance and withdrawal. With that said, it is impossible in our society to make all things equal. There are clear pharmacological differences in what happens in the brain and the body that contribute to what we are calling addiction. However, these cannot be readily separated in our society. Access, dose, route of administration, societal acceptance, perceived risk, cost, societal consequences for use or intoxication, and multiple other factors contribute to the real-world question of how addictive a drug is compared to another.

**Dr. Margaret Haney:** Let me add that although it is correct that our numbers with relapse are very high, we have to be aware that this could also reflect the permissive attitude toward Cannabis. My impression is that patients coming in for treatment have not reached the point that many cocaine users and opiate users do, where they have to stop because they have hit the proverbial rock bottom. Cannabis users are a bit more ambivalent about quitting so that could feed into the high relapse rates.

The overall point is that Cannabis is not a thoroughly benign drug. Cannabis is not the worst drug, but it is not a drug without consequences. Again, societal attitudes often seem to skew one way or the other; it is all good or it is all bad, when it is clearly both.

**Dr. Daniele Piomelli:** **Great way of putting it, Dr. Haney. Looking ahead, I have a few questions about ways to treat CUD. First of all, should we treat it? Is it something that needs to be treated? Dr. Haney, what do you think?**

**Dr. Margaret Haney:** Yes, 100%. As Cannabis researchers and clinicians, we want to have a range of options available for people seeking treatment. Some patients are going to prefer a behavioral, psychological treatment approach. I strongly believe we also have to provide patients with the option of a pharmacological treatment approach and let the patients choose what works for them. We want to have a range of options available to help somebody once he or she decides to quite Cannabis use.

**Dr. Alan J. Budney:** I would just add that, because such ambivalence and ambiguity about Cannabis exists and the problems it might cause, we should not neglect the need for early interventions or preventative interventions that educate and motivate individuals to watch out and perhaps make changes to their Cannabis use patterns before problems develop or move from mild to moderate or severe level. So, I guess you might label that a treatment option, but perhaps more accurately, a preventative option should also be on the table.

**Dr. Margaret Haney:** Right, education includes having an honest discussion about the risks and harms without exaggeration but with presenting the real risks.

**Dr. Alan J. Budney:** One of the issues with our conversation so far is that it has been highly skewed toward discussing problems and consequences. We did not talk about the probability of developing a problem once you try Cannabis, which, like other drugs, the majority of people that use Cannabis do not go on to have problems. I think it is very important to make such a point clearly and repeatedly during these types of conversations. The reason being that I think it helps the educational process by allowing more people to listen and perhaps accept and consider the possibility that many people can develop real and substantial problems related to the use and misuse of Cannabis.

**Dr. Daniele Piomelli:** **I would like to go back to the issue of treatment. What options are available to treat Cannabis addiction right now? If I were to show up at your hospital, Dr. Haney, and say, “I have got a problem with Cannabis,” what could you do for me?**

**Dr. Margaret Haney:** Right. Well, my colleagues would enroll you in a clinical trial where we would both administer behavioral treatments and test a potential medication. There is no FDA-approved medication at this moment. At Columbia University what we are doing is clinical trials, testing things that have looked promising in the laboratory and moving them into the clinic. Dr. Budney has extensive experience with behavioral treatment options and some pharmacology as well, and so he treats more patients than I do. Alan?

**Dr. Alan J. Budney:** Yes, we would provide the same types of treatment that Dr. Haney just mentioned. The behavioral treatment options are pretty much the same as those used with any other substance use disorder. There are cognitive behavioral therapies that have been well specified. There are motivational interventions that are well specified. There are incentive-based or contingency management-based interventions that are well specified, with different intensity levels depending on the magnitude or the severity of the problem.

Again the option of combining these behavioral treatments with medications is always an important consideration. Currently, some providers use medications that are not FDA approved specifically for CUDs, and much of this practice is to target symptoms such as nausea, depressed mood, insomnia, or appetite loss, which are common symptoms experienced during the early withdrawal phase immediately after cessation. However, that is an individualized practice, and certainly not a standard part of therapies for CUD.

**Dr. Pier Vincenzo Piazza:** To give a perspective from the other side of the ocean, the European Monitoring Center for Drugs and Drug Addiction (EMCDDA) has published this year a very well-done analysis of all the available behavioral treatments for Cannabis. This analysis compares different behavioral therapies, and in particular the ones that are generic for all drugs to the ones that are specific for Cannabis abuse.^2^

All the therapies were plus or minus effective showing a small but significant effect. However, there was no difference between the Cannabis-specific approach and the approaches that are designed for treating drug use disorders in general. One important point that has been unanimously underlined by behavioral therapists, at a convention on CUDs organized by the Swedish minister of health, is that patients with CUD are particularly difficult to treat. They usually forget the previous session of therapy, and so each day you start all over again, and they suffer from a profound lack of motivation that makes them quite difficult to engage in the therapeutic process. This is due to the pharmacological effects of THC that impair memory and motivation.

So I think that, like for the other drugs and in general for behavioral diseases, it is really important to develop a pharmacological treatment of CUD.

**Dr. Daniele Piomelli:** **But we do not currently have pharmacological treatments?**

**Dr. Pier Vincenzo Piazza:** No, there is nothing that is approved by the FDA or EMEA. I am not aware of anything that has shown particularly strong effect.

**Dr. Margaret Haney:** In our laboratory model of CUD, we have found that the long-acting cannabinoid agonist, nabilone, has shown promise. Better than all the medications we have tested, nabilone has both reduced Cannabis withdrawal symptoms and reduced relapse as measured in the laboratory. However, these laboratory findings need to be confirmed in a clinical trial.

**Dr. Alan J. Budney:** Oh, yeah?

**Dr. Margaret Haney:** It is much better than dronabinol.

**Dr. Daniele Piomelli:** **If I take a poll of this small group of scientists, do you think that we should stop looking for treatments, pharmacological treatments for Cannabis abuse, or do you think we should move forward?**

**Dr. Alan J. Budney:** I'm afraid we are all quite biased in regard to this question, given that much of our life's work is devoted to trying to develop more effective ways to help people with problems to stop using or reduce their substance use.

**Dr. Margaret Haney:** I will commit hara-kiri if we stop.

**Dr. Alan J. Budney:** What we have been talking about, CUD, is very difficult to treat. We do not have high success rates with any of our most potent therapies. So, coming up with pharmacological and behavioral combinations is essential. Our current behavioral treatments do help many people with problems, but there are many that we leave behind. Clinical scientists are clearly making incremental progress in developing more potent behavioral approaches. Pharmacological possibilities are growing as we learn more about Cannabis and its pharmacological actions. Definitely, both approaches should continue to be pursued.

**Dr. Pier Vincenzo Piazza:** And I would like to add that scientific knowledge on the cannabinoid system and on the CB1 receptor, the principal target of THC, has progressed very much during the last 20 years. It is probably one of the biological systems that we know best, and we know more about it every day. The scientific community should now start looking for a treatment of CUD. I do not think scientists have done very much to find treatments. Myself, I have started doing it, but I think that other people should get into the game.

I really believe that if we put some serious effort into the research, then we will be able to develop a true treatment for CUD. Substitution treatments like nabilone could be useful but are far from ideal. Having an analog of THC constantly on board can be associated with serious health problems such as cardiovascular risk, cognitive impairments, and an increased risk of fibrosis.

**Dr. Daniele Piomelli:** **I am sorry, but we are out of time. This was a wonderful conversation. If you have any last words of wisdom you would like to add, it would be great to hear them.**

**Dr. Margaret Haney:** Well, just to follow up on Dr. Piazza, it is an exciting time in the cannabinoid field because we are really in its infancy. It was not until the 1990s that we really started moving on the endocannabinoid system, as you all know very well, and so there is a tremendous amount of work to be done.

**Dr. Daniele Piomelli:** **Thank you very much, everybody!**
